# Local structure of molten AuGa_2_ under pressure: Evidence for coordination change and planetary implications

**DOI:** 10.1038/s41598-018-25297-9

**Published:** 2018-05-01

**Authors:** Bora Kalkan, Budhiram Godwal, Selva Vennila Raju, Raymond Jeanloz

**Affiliations:** 1 Department of Physics Engineering, Hacettepe University, Beytepe, 06800 Ankara, Turkey; 2 Advanced Light Source, Lawrence Berkeley National Laboratory, Berkeley, CA USA; 3 Department Earth and Planetary Science, University of California, Berkeley, CA USA; 4 Presently at Oakridge Associate Universities, Belcamp, MD 21017 USA; 5 Department Astronomy and Miller Institute for Basic Research in Science, University of California, Berkeley, CA USA

## Abstract

*In situ* x-ray diffraction measurements and inverse Monte Carlo simulations of pair distribution functions were used to characterize the local structure of molten AuGa_2_ up to 16 GPa and 940 K. Our results document systematic changes in liquid structure due to a combination of bond compression and coordination increase. Empirical potential structure refinement shows the first-neighbor coordination of Ga around Au and of Au around Ga to increase from about 8 to 10 and 4 to 5, respectively between 0 and 16 GPa, and the inferred changes in liquid structure can explain the observed melting-point depression of AuGa_2_ up to 5 GPa. As *intermetallic* AuGa_2_ is an analogue for *metallic* SiO_2_ at much higher pressures, our results imply that structural changes documented for *non-metallic* silicate melts below 100 GPa are followed by additional coordination changes in the metallic state at pressures in the 0.2–1 TPa range achieved inside large planets.

## Introduction

The study of densified liquid structures provides fundamental information for understanding thermodynamic phase diagrams, including the effects of distinct crystalline phases on melting at various pressures and temperatures. The intermetallic compound AuGa_2_ exhibits rich polymorphism in the solid state^1–3^, transforming from 8- to 10-coordinated crystal structures (Ga around Au) at pressures below 30 GPa. We present high-pressure x-ray diffraction measurements in order to determine whether analogous structural changes take place in the liquid. The melting temperature of AuGa_2_ is known to go through a minimum around 5.5 GPa^3^, presumably because of competing structural changes in the crystalline and liquid phases, but this interpretation needs verification through measurements on the melt under pressure. Developments in experimental techniques over recent years are providing rich information about liquid structures over a much wider range of pressures than heretofore possible, complementing the vast amount of data available for crystal-structural transformations under pressure^4–6^. Moreover, there is only limited understanding of the local structure of solid amorphous AuGa_2_, with the available information amounting to the total structure factor obtained from electron diffraction^7^, which does not provide such detail as pair distribution functions from experiment or modelling (e.g., Monte-Carlo modelling).

## Results from Experiments and Modeling

**Figure 1:**
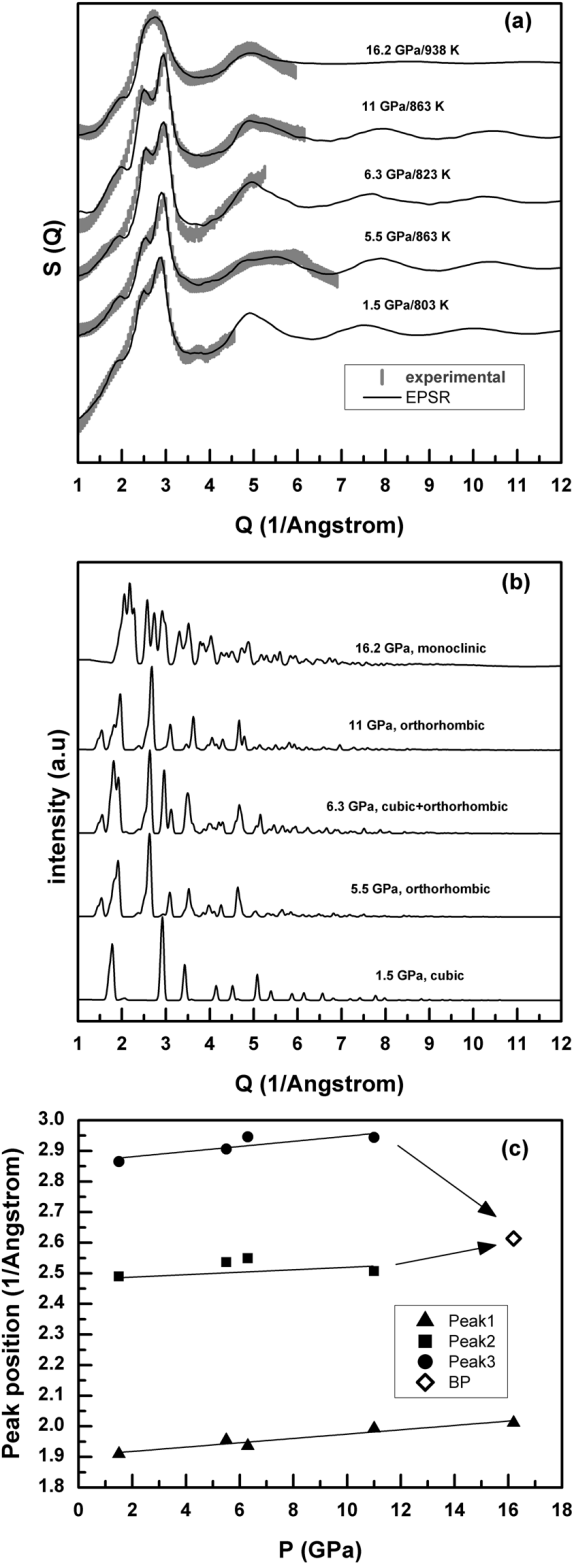
(**a**) Experimental (thick grey lines) and EPSR simulated (thin black lines) total structure factors, *S*(*Q*), for liquid AuGa_2_ at different pressures. (**b**) Diffraction patterns for crystalline phases at corresponding pressures. (**c**) Pressure dependence of the liquid *S*(*Q*) peaks for 1.5 < *Q* < 3.5 Å^−1^, with Peaks 2 and 3 merging to form a broad peak (*BP*) at 16 GPa.

Data were obtained by heating each of the crystalline phases of AuGa_2_ to temperatures slightly above melting (Table 1), the two-dimensional diffraction patterns recorded by image plate confirming the absence of any crystalline phase after melting^3^. In spite of our limited *Q* range, which constrains the simulation of measurements to low magnitudes of the scattering vector *Q*, we were able to observe distinct features in the diffraction patterns for liquid AuGa_2_ at pressures at which different sub-solidus crystal structures are stable (Table 2, Fig. 1a).

**Table 1:** Pressures measured during heating for individual experimental runs, with C, O and M representing cubic (fluorite), orthorhombic (cotunnite) and monoclinic (“post-cotunnite”) phases.

Run #1			Run #2			Run #7			Run #8			Run #9		
P(GPa)	T(K)	Phase	P(GPa)	T(K)	Phase	P(GPa)	T(K)	Phase	P(GPa)	T(K)	Phase	P(GPa)	T(K)	Phase
0	295	C	0	295	C	0	295	C	0	295	C	0	295	C
1.5	295	C	7.7	295	C, O	8.8	295	O	10.7	295	C, O	16.2	295	C, M
1.5	473	C	6.3	673	O, C	8	395	O	11	723	O, C	16.2	673	M
1.65	743	C	6.3	723	O	5.5	763	Melt, C	11	803	Melt, O, C	16.2	773	M
1.5	763	C	6.3	773	Melt, C	5.5	773	Melt	11	863	Melt	16.2	930	Melt
1.5	783	C	6.3	783	Melt									
1.5	803	Melt												

Uncertainties in pressure and temperature are up to ±2 GPa and ±5 K.

**Figure 2:**
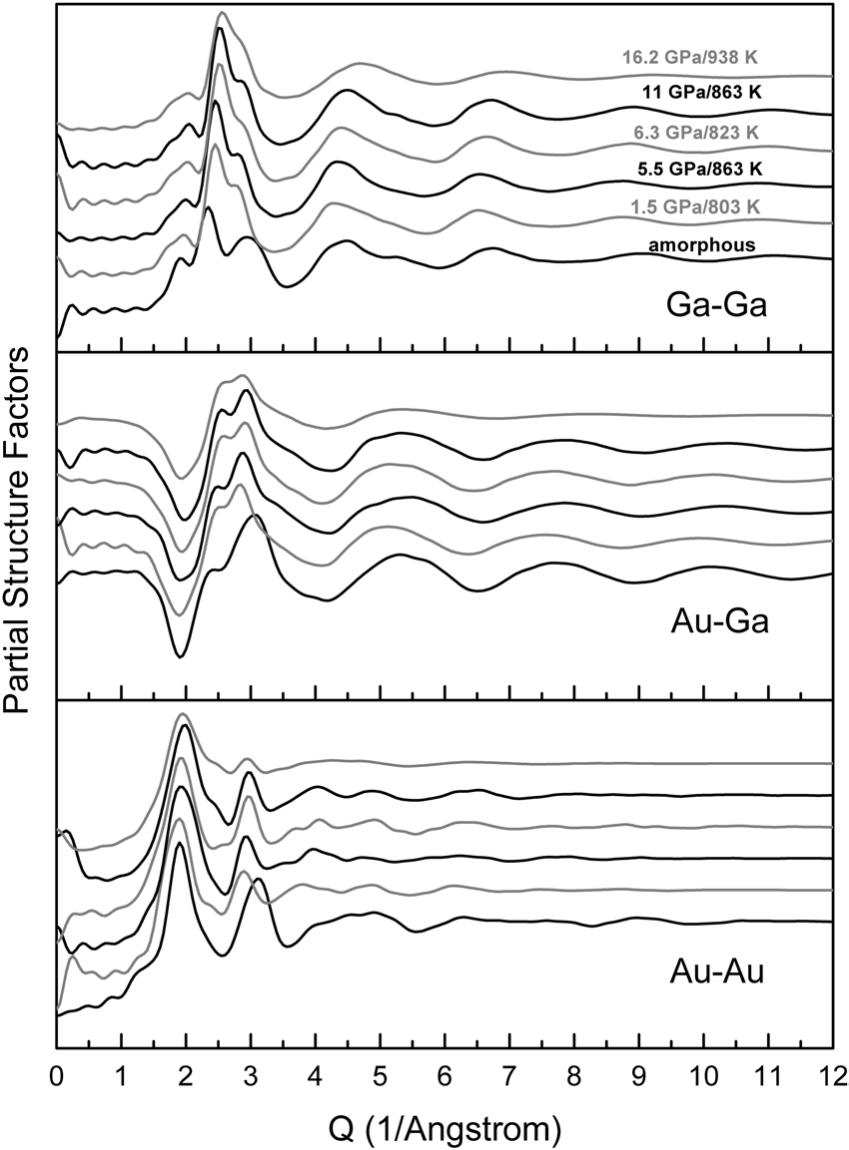
Calculated partial structure factors for amorphous (*P* = 0, *T* = 300 K) and liquid AuGa_2_ under pressure. Data are vertically offset for clarity.

The most prominent peak (Peak 3, Fig. 1c) in the structure factor *S*(*Q*) at temperatures above the melting temperatures of cubic and orthorhombic phases (*P* ≤ 11 GPa) has a clear shoulder on the low-*Q* side (Peak 2, Fig. 1c). This is distinct from the result for the monoclinic-to-melt pattern (*P* = 16 GPa, Fig. 1a), which shows a broad peak at *Q* = 2.62(5) Å^−1^ attributable to overlapping of the prominent peak and shoulder. Peak 3 for the liquid *S*(*Q*) is in the range of 2.87–2.95 Å^−1^ at 11 GPa and below, matching the highest peaks in the diffraction patterns of the three crystalline phases (Fig. 1b). Partial structure factors calculated from our simulations show that Au-Au correlations give the major contribution to the first sharp diffraction peak (Peak 1) in *S*(*Q*); Peak 2 arises from Ga-Ga correlations, along with smaller contributions from Au-Ga correlations; and Peak 3 can be attributed to Au-Ga and Au-Au correlations (Fig. 2). Qualitatively, the increases in *Q* values for Peaks 1–3 to 11 GPa are consistent with compaction of intermediate-range order in the liquid, whereas evolution of the main peak into a broad maximum at *Q* ~ 2.6 Å^−1^, similar to the value at zero pressure, suggests an increase in first-neighbor coordination that is causing an increased bond length as the sample is compressed.

For purposes of model validation, we have also considered the amorphous (glass) phase of AuGa_2_ at *P* = 0 GPa and *T* = 300 K based on data of Bohórquez^7^. In general, the oscillations in *S*(*Q*) for liquid AuGa_2_ under pressure have a lower amplitude and die away more rapidly with *Q* than do those for the amorphous phase at ambient conditions, while the peak splitting in the 2.0–3.3 Å^−1^ range for molten AuGa_2_ becomes indistinct from the corresponding *S*(*Q*) range for amorphous AuGa_2_ (Supplementary Figure S8). The prominent Peak 3 is located at 3.08(5) Å^−1^ and 2.95(5) Å^−1^ (~4% change) for amorphous and orthorhombic-to-melt phases, respectively. The position of the shoulder on the low-*Q* side (Peak 2) is at 2.44(7) Å^−1^ in the *S*(*Q*) data obtained from the amorphous state, and changes to 2.51(8) Å^−1^ in the liquid at 11 GPa and 863 K, a ~3% change. The ratio of positions for Peak 3 and Peak 2 changing from ~1.26 at ambient conditions to ~1.17 at 11 GPa suggests increasing complexity in the structure of liquid AuGa_2_ under pressure. The first sharp diffraction peak (Peak 1) in *S*(*Q*) of amorphous AuGa_2_, at ~1.88 Å^−1^, increases steadily to ~2.01 Å^−1^ with pressure, corresponding to intermediate-range structural order at a length scale of ~ 3.2 Å (Fig. 1c and Supplementary Figure S11).

**Figure 3:**
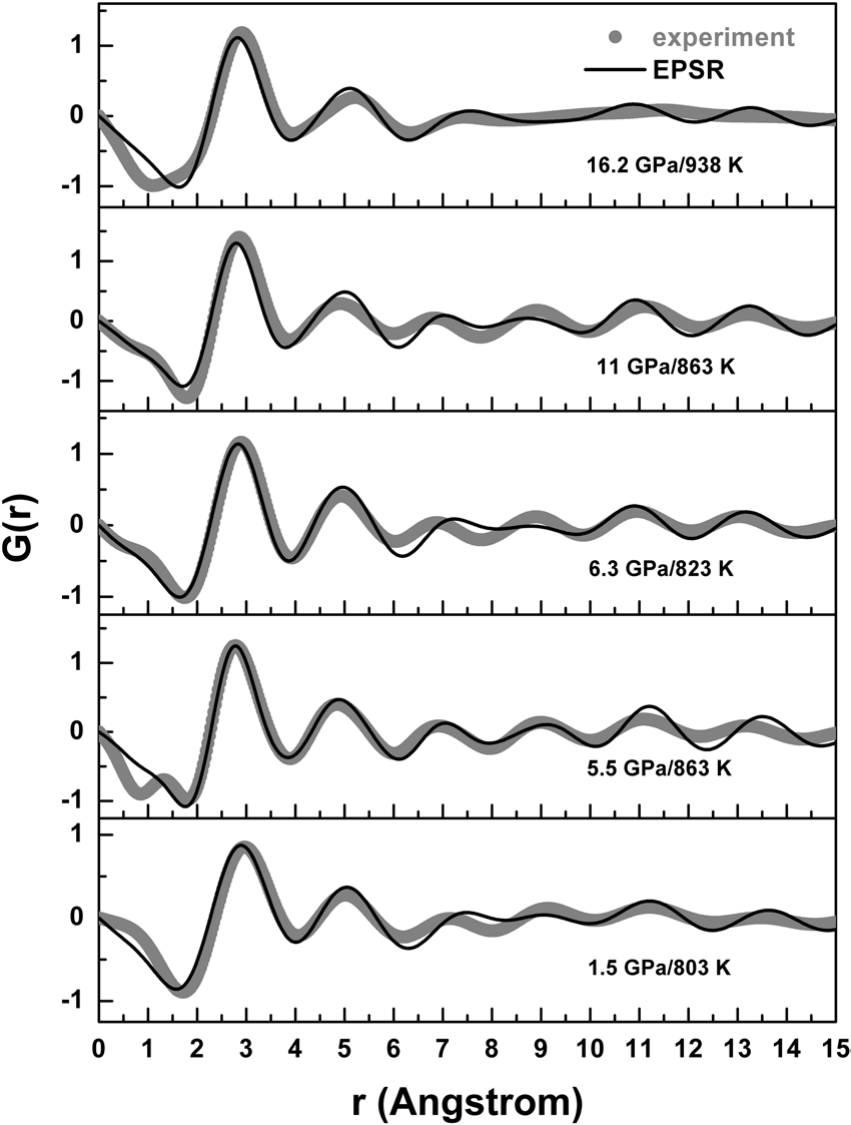
Calculated (lines) and experimental (grey dots) reduced pair distribution functions, *G*(*r*), for liquid AuGa_2_ under pressure.

**Figure 4:**
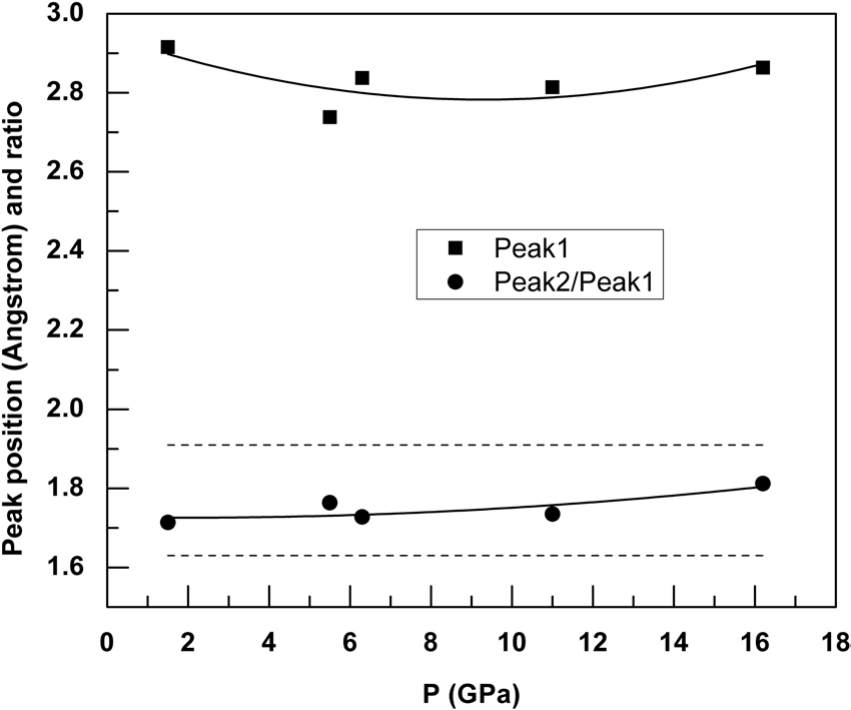
Positions of the first peak in the reduced pair distribution functions, *G*(*r*), for liquid AuGa_2_ (*squares*) and ratio of the second to first peak positions (*circles*). The dashed lines indicate the ideal ratio determined for a simple close-packed hard sphere-like structures (*r*_2_/*r*_1_ ≈ 1.91) and for an ideal tetrahedral structural unit (*r*_2_/*r*_1_ ≈ 1.63)^8^. The errors in peak positions are in the range of ±0.002–0.018, smaller than the corresponding symbols.

We test the idea of increased liquid-structure complexity under pressure by deriving the reduced pair distribution function (PDF) *G*(*r*) for the liquid: it has a strong peak at 2.73 to 2.91 Å with increasing pressure and a secondary peak at 4.82 to 5.19 Å over the 0–16 GPa pressure range (Figs 3 and 4), providing a good fit to the experimental data (accurate representation of short range structure) up to ∼6 Å at each pressure point. The differences between calculated and experimental *G*(*r*) above ∼6 Å indicate that intermediate range order in liquid AuGa_2_ is not well represented, however. This is a subject for more detailed PDF analysis using diffraction data collected with higher-energy x-rays, but does not affect the present interpretations.

**Table 2:** Pressures, temperatures and initial atomic number densities used for EPSR simulations.

P (GPa)	T(K)	Crystal phase-to-melt	Au Coordination (*n*_*AuGa*_)	Atomic number density (atoms/Å^3^)
0	300	Cubic fluorite-type	8	0.0535*
1.5	803	Cubic fluorite-type	8	0.0547
6.3	823	Orthorhombic+Cubic	8–9	0.0577
5.5	863	Orthorhombic cotunnite	9	0.0571
11	863	Orthorhombic cotunnite	9	0.0595
16.2	938	Monoclinic “post-cotunnite”	~10	0.0615

^*^Amorphous (glass) state at ambient conditions: data from literature^7^.

**Figure 5:**
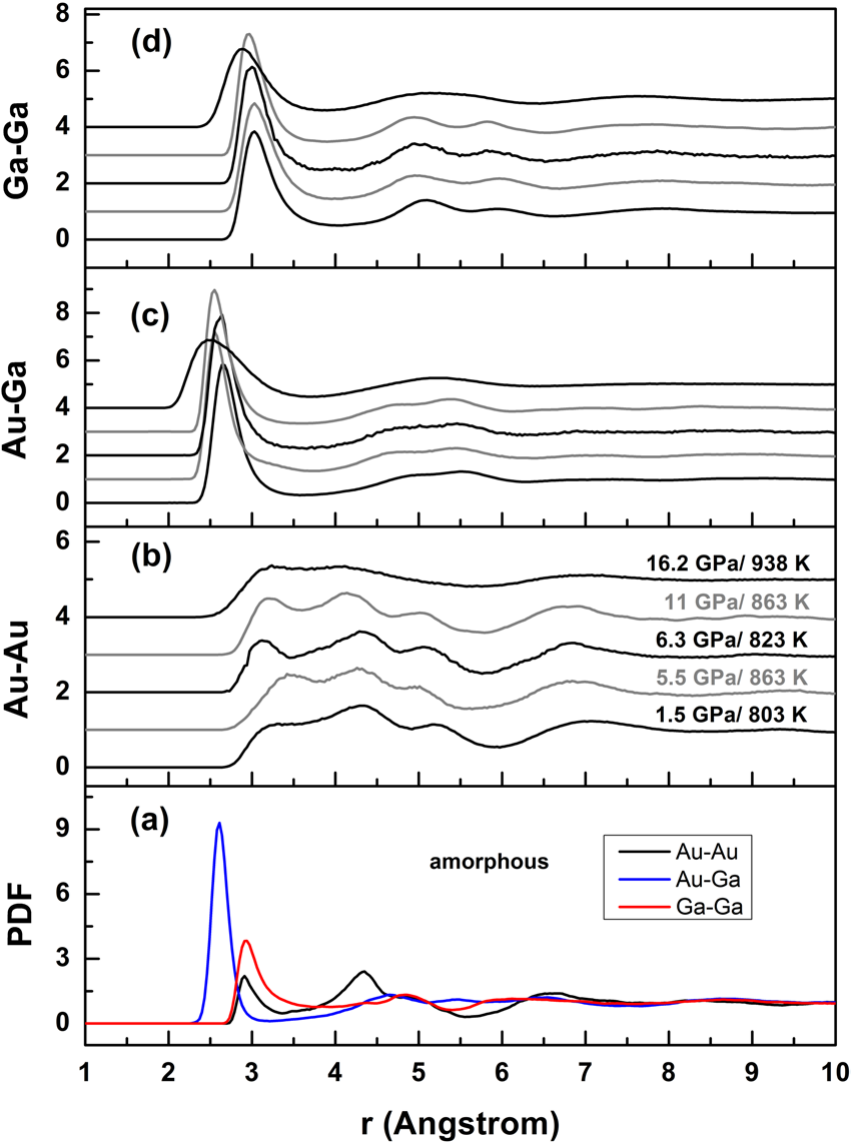
Au-Au, Au-Ga, and Ga-Ga partial pair distribution functions calculated from the EPSR simulations for (**a**) amorphous AuGa_2_ at *P* = 0 and *T* = 300 K, and (**b**,**c**,**d**) liquid AuGa_2_ under pressure.

**Figure 6:**
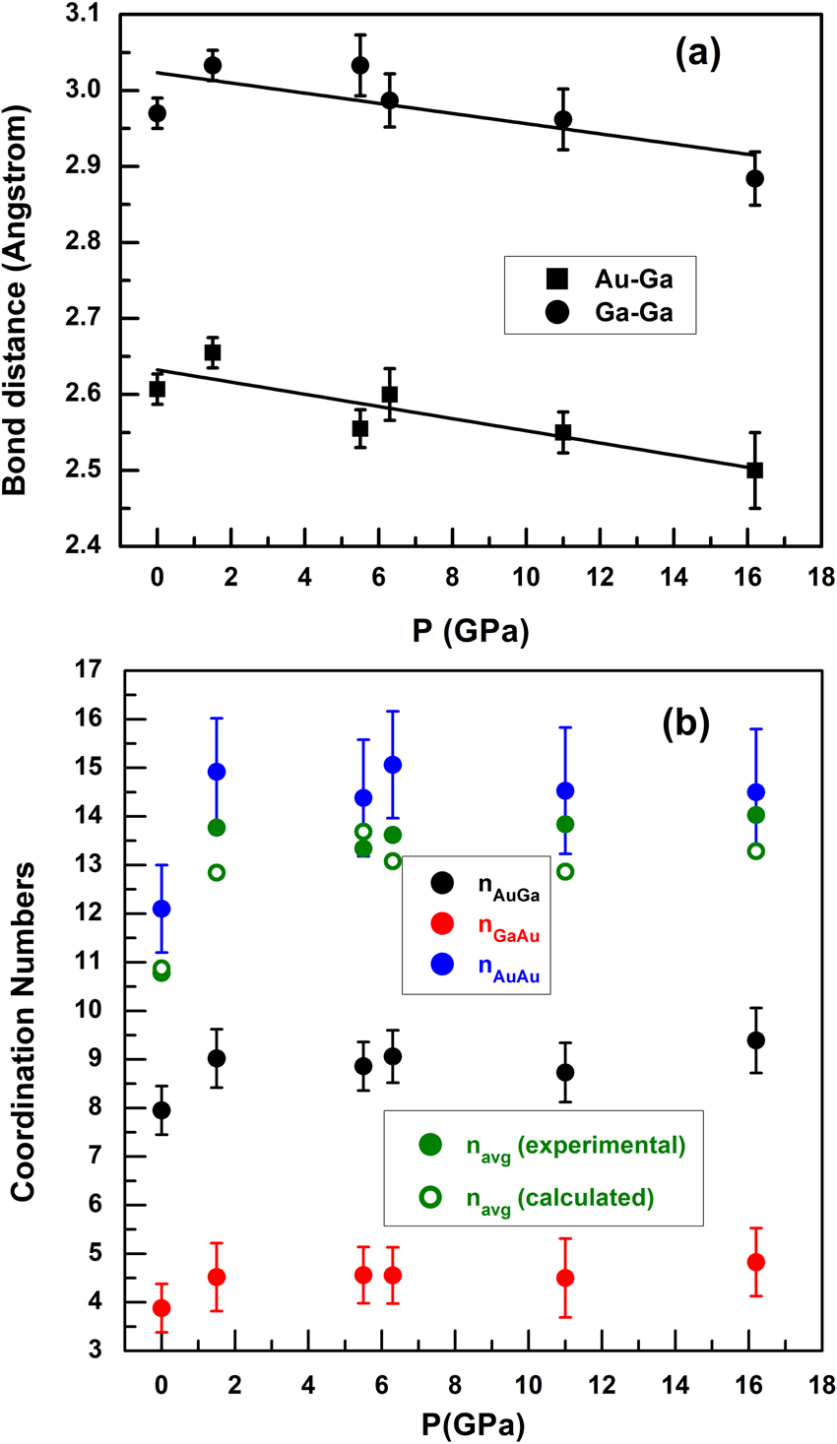
(**a**) Pressure dependence of Au-Ga (nearest-neighbor) and Ga-Ga bond distances determined from partial pair distribution functions (Fig. 5). (**b**) Coordination numbers around Au- and Ga-atoms, *n*_*AuGa*_, *n*_*GaAu*_ and *n*_*AuAu*_, and experimental and calculated average coordination numbers.

The ratio of second to first neighbor distances shifts from 1.67 to 1.81 with increasing pressure, neither value being close to that expected for simple hard sphere-like structures (for which *r*_2_/*r*_1_ ≈ 1.91). The Au−Au, Au−Ga and Ga−Ga pair distribution functions, obtained from Fourier transforming the corresponding partial structure factors, show that the main peak in *G*(*r*) (1.8 < *r* < 4.0 Å) has contributions from first-neighbor Au-Ga for *r* < 2.8 Å and second-neighbor Ga-Ga and Au-Au correlations for *r* > 2.8 Å (Fig. 5). The Au-Ga and Ga-Ga nearest-neighbor distances are calculated to be in the range 2.50–2.66 Å and 2.88–3.03 Å, respectively, decreasing ∼4.5% and ∼4.8% over our pressure range (Fig. 6a). The pressure-induced broadening observed for the first Au-Ga peak can be associated with a change in coordination number around Au, and the pressure dependence of the Ga-Ga pair distribution function is similar to that of Au-Ga; the Au-Au pair distribution functions have larger statistical uncertainty than for Au-Ga and Ga-Ga, due to the smaller relative fraction of Au atoms in the AuGa_2_ stoichiometry.

## Coordination Changes and Discussion

Nearest-neighbor coordination numbers around Au- and Ga-atoms, *n*_*AuGa*_, *n*_*GaAu*_ and *n*_*AuAu*_, obtained by integrating the areas of the corresponding peaks in the *g*_*AuAu*_(*r*) and *g*_*AuGa*_(*r*) pair distribution functions (Table 3), show systematic increases with pressure (Fig. 6b). The results at zero pressure are consistent with eight-fold coordination of Ga around Au in the liquid, the coordination in the cubic CaF_2_ crystal structure that is stable at ambient conditions. However, our model indicates that the first-neighbor coordination increases rapidly in the liquid, toward nine-fold by 1–5 GPa, implying a denser melt relative to the coexisting crystal structure, as documented by the negative Clapeyron slope for the melting temperature^3^. That is, the pressure-induced melting-point depression for AuGa_2_ at low pressures, with a turnaround at 5 GPa, can be explained in terms of changes in liquid and crystal structures.

**Table 3:** The details of integrating procedure at coordination numbers calculation.

r_min_ (Å)	r_max_ (Å)	Coordination number	Pair distribution function	Figure#
1.0	3.3	*n*_*AuGa*_	*g*_*AuGa*_(*r*)	5c
1.0	3.3	*n*_*GaAu*_	*g*_*GaAu*_(*r*)	5c
2.0	5.7	*n*_*AuAu*_	*g*_*AuAu*_(*r*)	5b
1.0	3.8	*n*_*Avg*_ (calculated)*	*g*_*AuGa*_(*r*), *g*_*AuAu*_(*r*), *g*_*GaAu*_(*r*), *g*_*GaGa*_(*r*)	5a, 5b, 5c
1.0	3.8	*n*_*Avg*_ *(experimental)*	*ρ*_0_ 4*πr*^2^*g*(*r*)	S12**

^*^n_*Avg*_ (*calculated*) = *C*_*Au*_ [*n*_*AuGa*_ + *n*_*AuAu*_] + *C*_*Ga*_ [*n*_*GaAu*_ + *n*_*GaGa*_], *C*_*Au*_ ≈ *0.33* (Atomic concentration of Au) and *C*_*Ga*_ ≈ *0.67* (Atomic concentration of Ga).

**See Supplementary Figure S12.

With rising pressure and temperature, we find *n*_*AuGa*_ gradually increasing to 9.4(7) at 16.1 GPa, implying nine- and ten-fold coordination around Au in the melt above 15 GPa. Given the stoichiometry of AuGa_2_, we expect the coordination of Au around Ga to correspondingly rise from about 8/2 = 4 at zero pressure to 10/2 = 5 at 15 GPa, which is consistent with the calculated average value of *n*_*GaAu*_ that increases from 3.9(4) to 4.9(5) at 16.2 GPa (Fig. 6b). The presence of GaAu_4_ structural units in the melt at low pressure is also in line with the ratio of second to first neighbor distances observed in *G*(*r*), *r*_2_/*r*_1_ ≈ 1.63 for tetrahedra (Fig. 4)^8,9^. Integration of the broad peak in the Au-Au pair distribution function (3 < *r* < 6 Å) gives a coordination number *n*_*AuAu*_ = 12.1(8) at ambient conditions and rising to 14.5(9) by 16 GPa, confirming that liquid AuGa_2_ densifies via collapse of the second- and higher-coordination shells under pressure. We find that the average coordination number calculated over the range 1.0 < *r* < 3.8 Å, rises from *n*_*average*_ = 10.8(6) at ambient conditions to 13.3(7) 16 GPa based on the fits to our experimental data, and this compares well with *n*_*average*_ calculated by integrating the intensity under the peak in *g*(*r*), *ρ*_0_ 4*πr*^2^*g*(*r*) (Fig. 6b and see Supplementary Figure S12).

**Figure 7:**
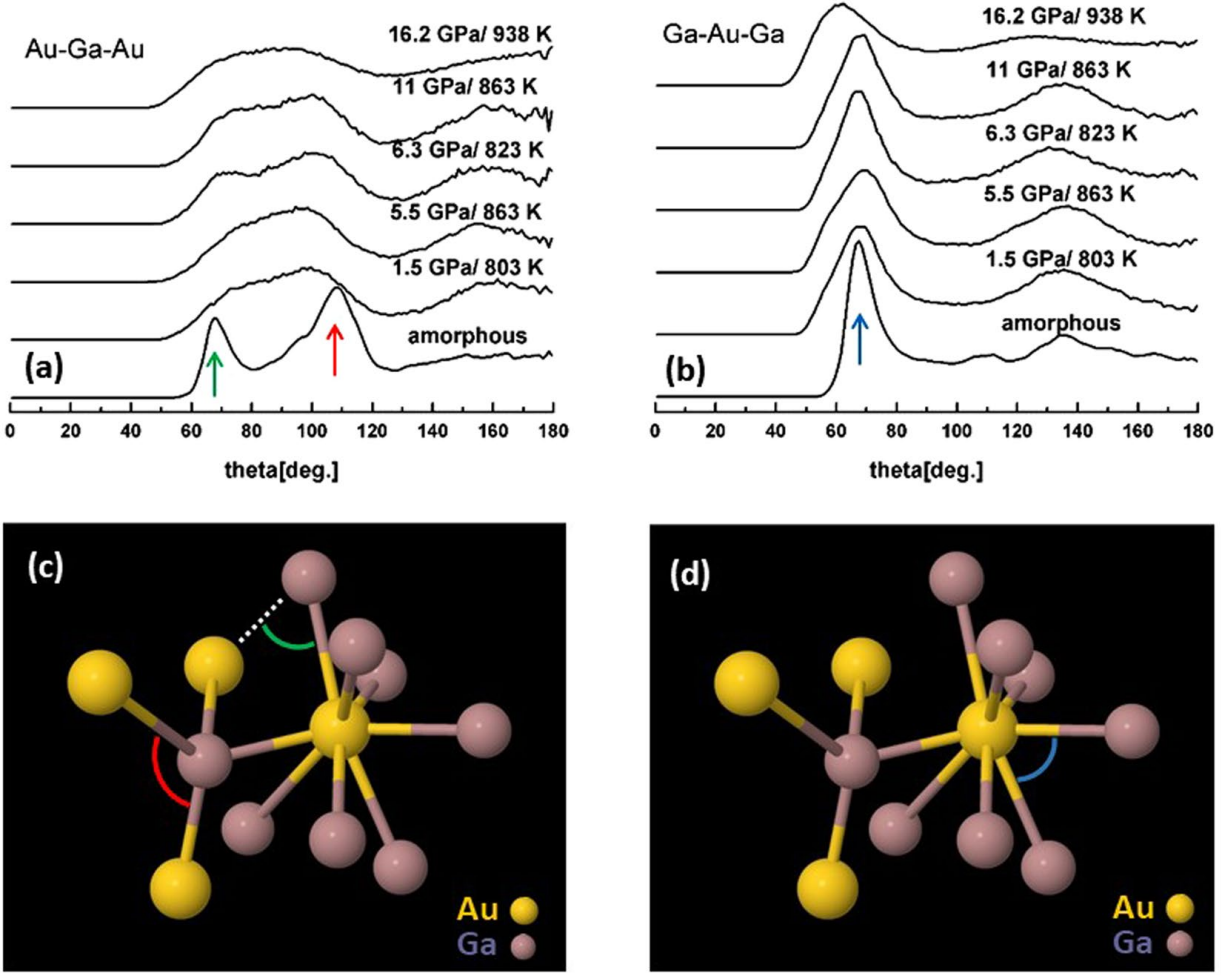
Au-Ga-Au (**a**) and Ga-Au-Ga (**b**) bond-angle distributions as functions of pressure, and structural motifs at zero pressure (**c**,**d**) captured from EPSR.

**Figure 8:**
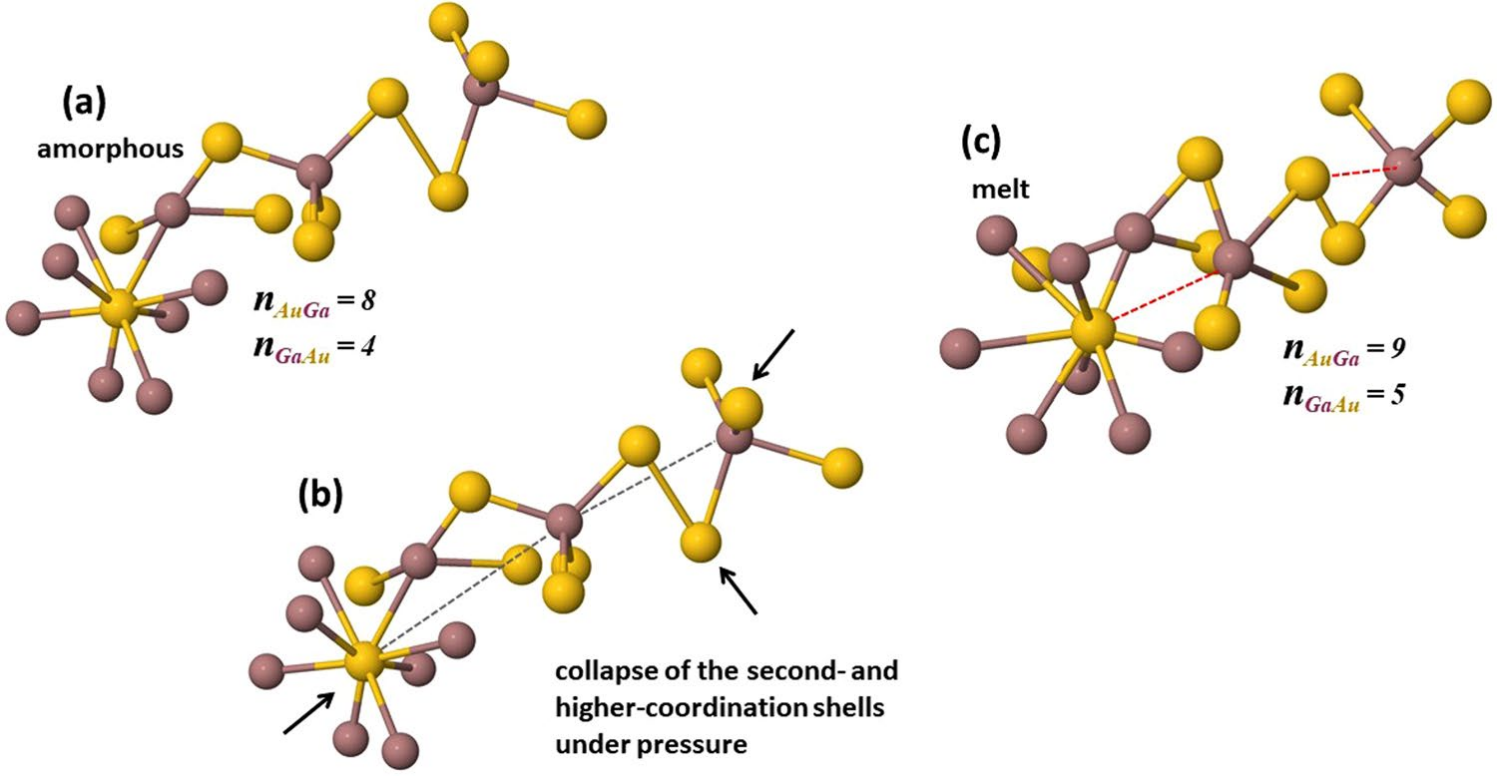
Schematic representation of densification mechanism and corresponding structural moieties in liquid AuGa_2_. Amorphous solid (**a**) includes GaAu_4_ tetrahedral units connected to each other via Au-Au bonding and to AuGa_8_ polyhedral units via Au-Ga bonding. Densification is dominated by large inward shifting of the second- and higher coordination shells under pressure (**b**). Melt-AuGa_2_ obtained at highest pressure shows higher order polyhedral units, 5 and 9 coordination around Ga and Au atoms, respectively (**c**).

Ga-Au-Ga and Au-Ga-Au bond-angle distributions were calculated for neighbors separated by less than 3.3 Å. The bond-angle distributions obtained from amorphous-AuGa_2_ at ambient conditions are characterized by peaks at ∼68° and 108° for Ga-Au-Ga and Au-Ga-Au, close to the ideal values for 8-fold and tetrahedral (109°) coordination and consistent with our estimates for *n*_*AuGa*_ and *n*_*GaAu*_, respectively (Fig. 7). With increasing pressure, the Au-Ga-Au angle distribution forms a broad peak centered near 86° that accords with fivefold coordination. The main Ga-Au-Ga distribution peak broadens and becomes asymmetric with pressure, indicating coordination change toward a distribution of first-neighbor configurations ranging from 7.9 to 9.4 (Fig. 8).

Silica is the archetypal rock-forming compound making up terrestrial planets, and it is expected to take on the cotunnite structures and to become metallic at multi-Mbar (~200–500 GPa) pressures^1,10–16^. Therefore, we expect that fluid AuGa_2_ may serve as an analog of the liquid metallic silicates present at the high pressures relevant to the interiors of large planets, such as super-Earths and other extrasolar planets^17^. Moreover, it has been found that silicate melts (magmas) undergo structural transformations, with increasing first-neighbor coordination reducing the volume difference between crystal and melt, such that magmas can sink at depth rather than being buoyant as observed at the surface (e.g., causing volcanic eruptions)^17,18^. These conclusions have been documented through high-pressure spectroscopy and x-ray diffraction on silicate (and analogue) melts and glasses, documenting Si-O coordination increasing from 4 to 6 by about 35 GPa^19–24^; recent diffraction results show no significant coordination increase beyond 6–6.8 to nearly 200 GPa^25^, however. Along with flattening of the melting curve, *T*_*m*_(*P*), atomistic models predict that the adiabatic temperature gradient (or Grüneisen parameter, *γ* = (∂ln*T*/∂ln*ρ*)_*S*_) of the melt exhibits the unusual tendency of *increasing* over a range of depths due to increasing coordination^26,27^ (*T*, *P* and *ρ* are temperature, pressure and density; subscripts *m* and *S* refer to melting and constant entropy).

Our results provide evidence for pressure-induced coordination change in intermetallic AuGa_2_, a potential analogue for the liquid metallic state of SiO_2_ that is stable at pressure relevant to large planetary interiors. Thus, based on our measurements we expect that the Si–O coordination in silicate melts increases beyond the 4- to 6-fold range documented by experiments to date up to 100–200 GPa, and toward 10-fold at pressures above 500 GPa where the cotunnite phase of SiO_2_ is predicted to be stable. Such changes in liquid structure are expected to affect planetary evolution by influencing the buoyancy of magmas relative to coexisting crystals over specific depth ranges within a planet, with element partitioning between liquid and crystals potentially determining whether melts sink or rise, and the Grüneisen parameter exhibiting anomalous pressure dependencies as at lower pressures.

## Methods

### Experimental study

AuGa_2_ was prepared by arc melting the elements under an Ar atmosphere, and x-ray diffraction confirms the expected cubic fluorite-type structure (*Fm3m*) with lattice parameter, *a* = 6.079(3) Å^28^. We used a resistively heated diamond-anvil cell (DAC) driven by a gas membrane for the present experiments^29^. A 10 × 10 µm beam of 25 keV energy (0.4959 Å wavelength) x-rays, as selected by a Si(111) double-crystal monochromator at the Advanced Light Source beamline 12.2.2, was used to collect diffraction patterns; the beam was focused at the sample position^30^. We used BN backing plates with different opening angles on the detector side, which allowed diffraction data to be collected up to a maximum scattering vector-magnitude of *Q* = 7 Å^−1^, corresponding to a resolution in radial distribution functions *Δr* ~ 0.740 (±0.045) Å. Diffraction patterns were obtained using a Mar345 image plate located 290.0 (±0.1) mm from the sample, and processed with fit2d and Celref^31,32^. On-line ruby (Al_2_O_3_:Cr^3+^) and samarium-doped strontium tetra-borate (SrB_4_O_7_:Sm^2+^) fluorescence were used to determine pressure of the hot sample (stimulated with an in-line 200 mW blue diode laser to increase the photon counts at high temperatures). Temperature was measured to an accuracy of ±5 K up to 900 K, using a K-type thermocouple next to the diamond culet^3,29^. Rhenium was the gasket material, and liquefied argon served as the pressure-transmitting medium^3^.

For each of our experiments, the sample was taken to the desired pressure and then put through a heating and cooling cycle; we observed consistent pressure drifts during each cycle, caused by thermal expansion of the diamond-cell components (pressure-temperature paths for five runs are given in Table 1). Complete melting of AuGa_2_ is identified by loss of long-range order, indicated by disappearance of x-ray diffraction peaks of the crystalline phase and a simultaneous increase in diffuse scattering over the entire 2*θ* range (raw melt data collected by heating various AuGa_2_ phases are shown in Supplementary Figs S1–S5)^3^. A new sample was reloaded after each thermal cycle, and diffraction patterns from melting various AuGa_2_ phases are illustrated in Fig. 1a. The experimental melt patterns shown in Fig. 1a were collected separately on a region free of gasket peaks, with longer exposure time in order to get good peak intensity relative to background.

### EPSR method to refine 3D structural model

McGreevy^33^ reviews a wide range of issues relating to the inversion of diffraction data to derive real-space functions. Although emphasizing reverse Monte Carlo methods, McGreevy also discusses empirical structure refinement (EPSR), and Soper^34^ provides further details. We use the EPSR method to fit and extrapolate to high *Q* the total structure factor *S*(*Q*) derived from each diffraction pattern (Fig. 1a), using 1000 Au and 2000 Ga atoms in the simulation box at conditions summarized in Table 2. The *S*(*Q*) data are then Fourier transformed to get radial distribution functions (see Supplementary Figure S6a). Application of EPSR requires that the sample density is known, which makes characterizing the liquid structures challenging. The density of molten AuGa_2_ was determined using two different methods: (i) from the slope of the reduced pair distribution functions, *G*(*r*), below 2.2 Å (see Supplementary Figure S6b); and (ii) from experimental measurements of the unit-cell volumes of the crystalline phases just below the melting temperature (Supplementary Figure S6c). The former includes truncation errors, so here we present results based on using the latter method (Table 2). Storm *et al*.^35^ have estimated the volume changes occurring on melting of AuGa_2_ by making use of low-pressure *dT/dP* data, and evaluated entropies of fusion based on the assumption that the addition of the entropies of fusion for the elements along with the entropy of mixing gives a reasonable estimate for the entropy of fusion of the compound. The decrease in volume on melting of AuGa_2_ is estimated to be 0.5 cc/mole. This results in approximately ≤1% change in volume on melting up to about 10 GPa, and justifies the use of crystalline-phase density just before melting to estimate the liquid density. Atomic number densities calculated using the two methods are compared in Supplementary Table S1. The Lennard-Jones potential-well depth and range for Au (Ga) were set to 0.1632 (0.1750) kJ/mol and 2.93 (1.60) Å, with initial parameters perturbed until we obtained a satisfactory fit to the diffraction data. Minimum approach distances of 2.77, 2.30 and 2.70 Å for, respectively, Au-Au, Au-Ga and Ga-Ga pairs are calculated using atomic radii of Au and Ga, and served as constraints in carrying out Monte Carlo simulations of the experimental *S*(*Q*). Corrections for background and Compton scattering, as well as normalization by the atomic form factors (Supplementary Figure S7) are used to obtain the structure factor from the diffraction data^36^. The EPSR simulations show good agreement with zero-pressure diffraction data for amorphous AuGa_2_ (Supplementary Figure S8).

Our fits to experimental *S*(*Q*) yield chi-square values in the range *χ*^2^ = 0.002–0.016 (Supplementary Figure S9), with convergence in ~2600 iterations and best fits achieved after ~8000 iterations of the EPSR steps (Supplementary Figure S10). There remain issues about the reliability of the simulations, but EPSR uses reasonable assumptions to produce close fits to the experimental diffraction data, and the results can be Fourier transformed to obtain the radial distribution functions. The derived structural information (e.g., coordination numbers and bond angle distributions) provides one means of assessing the quality of the results.

## Electronic supplementary material

Supplementary Information
