# Supplementary material for: Local structure of molten AuGa2 under pressure: Evidence for coordination change and planetary implications
Source: Sci Rep. 2018 May 1;8:6844. doi: 10.1038/s41598-018-25297-9 (PMC5931613; doi:10.1038/s41598-018-25297-9)
Supplement: Supplementary file 1 — Supplementary Information [file 41598_2018_25297_MOESM1_ESM.docx]

**Supplementary Information for**

**Local structure of molten AuGa_2_ under pressure: Evidence for coordination change and planetary implications**

Bora Kalkan^1,2,*^, Budhiram Godwal^3^, Selva Vennila Raju,^2,4^ and Raymond Jeanloz^3,5^

*^1^Department of Physics Engineering, Hacettepe University, Beytepe 06800, Ankara, Turkey.*

*^2^Advanced Light Source, Lawrence Berkeley National Laboratory, Berkeley, CA, USA*

*^3^Dept. Earth and Planetary Science, University of California, Berkeley, CA, USA.*

*^4^Presently at Oakridge Associate Universities, Belcamp, MD 21017, USA.*

*^5^Dept. Astronomy and Miller Institute for Basic Research in Science, University of California, Berkeley, CA, USA.*

*Corresponding author: Bora Kalkan (bkalkan123@gmail.com)*

**Contents**

- 2D diffraction patterns and relevant integration: Section 1 and Figures S1-S5
- Determination of atomic number densities: Section 2, Table S1 and Figure S6
- Data reduction to obtain S(Q): Section 3 and Figure S7
- Capability of EPSR technique and quality of *S*(*Q*) data for high-pressure melt; cross check with high-quality *S*(*Q*) data from amorphous AuGa_2_ at zero pressure: Section 4 and Figure S8
- More details about EPSR simulations: Section 5, chi-square values (Figure S9) and number of iterations (Figure S10).
- First sharp diffraction peak in S(Q) curves: Figure S11.
- Radial distribution functions, *g*(*r*) in spherical coordinates. Figure S12.

1. Experimental x-ray diffraction data

Raw x-ray diffraction data and integrated patterns collected on AuGa_2_ at various pressures during thermal cycling are shown in Figure S1 to S5. Experimental details are given in the main manuscript. We have marked Re gasket peaks in Figs. S1 to S5 with solid circles. The melt pattern in each run used for S(Q) determination was integrated with masking option in FIT2D to get clean data (more details below, in section 3).

**
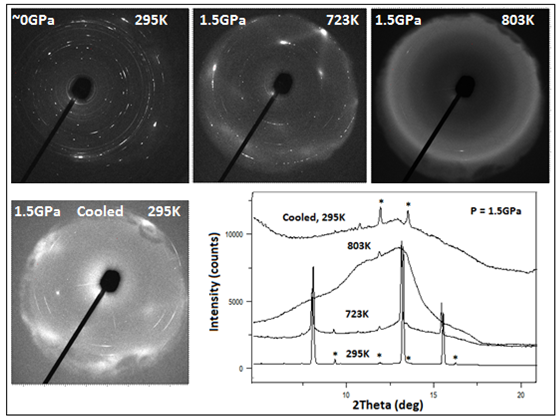
**

**Figure S1.** Raw x-ray diffraction data and stack plot (*lower right*) of Run #1 showing evolution of melting of AuGa_2_ starting from the cubic (CaF_2_) phase. The quenched sample shows a glassy structure. Solid circles denote peaks from rhenium gasket.


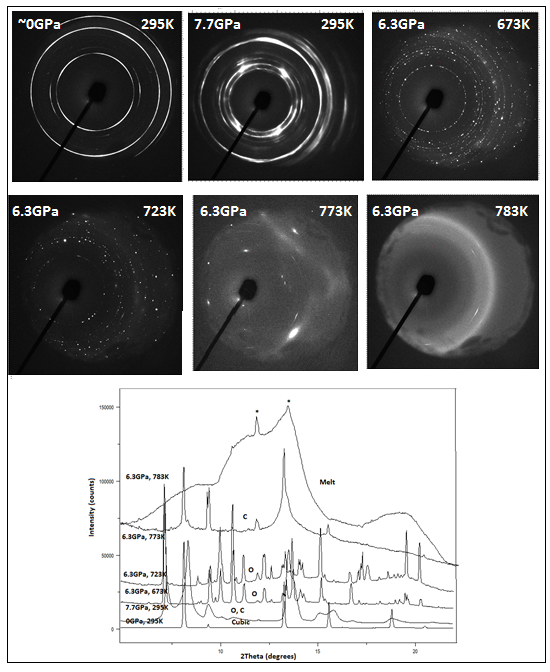


**Figure S2.** Raw x-ray diffraction data and stack plot (*bottom*) of Run #2 showing evolution of melting of AuGa_2_ starting from the orthorhombic (cottunite) and cubic phase at 7.7 GPa. Solid circles denote peaks from rhenium gasket.

**
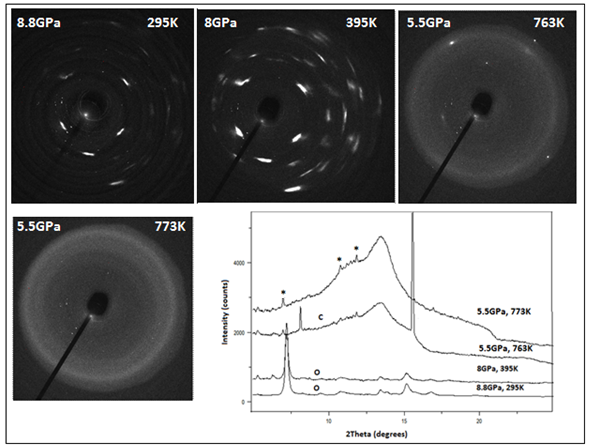
**

**Figure S3.** Raw x-ray diffraction data and stack plot (*lower right*) of Run #7 showing evolution of melting of AuGa_2_ starting from the orthorhombic phase at 8.8 GPa. Solid circles denote peaks from rhenium gasket.

**
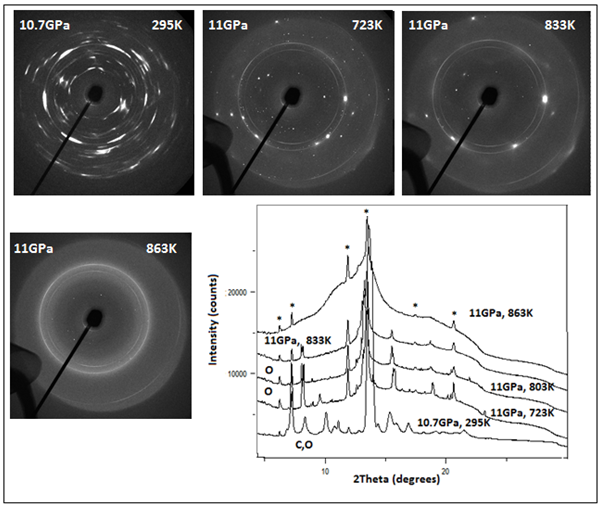
**

**Figure S4.** Raw x-ray diffraction data and stack plot (*bottom right*) of Run #8 showing evolution of melting of AuGa_2_ starting from cubic and orthorhombic phase at 10.7 GPa. No pressure drift was observed during this run before melting. Solid circles denote peaks from rhenium gasket.


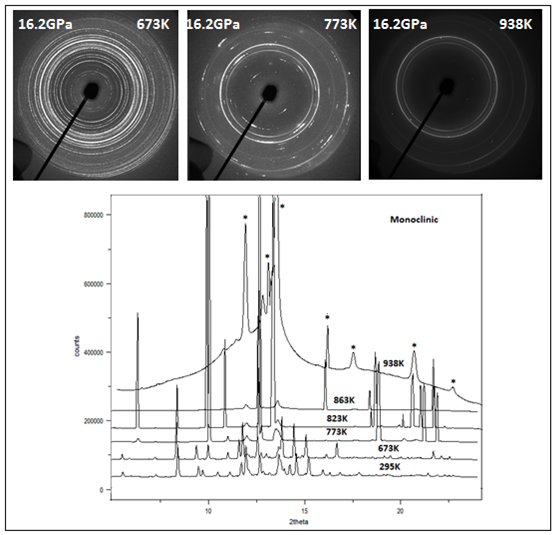


**Figure S5.** Raw x-ray diffraction data and stack plot (*bottom*) of Run #9 showing evolution of melting of AuGa_2_ starting from the monoclinic (“post-cotunnite”) phase. No pressure drift was observed during this run before melting. Solid circles denote peaks from rhenium gasket.

1. Determination of atomic number densities

Density values listed in the main text were determined using two different methods. The first approach used the slope of the atomic distribution [1] function below 2.2 Å. The *S*(*Q*) data were Fourier transformed to get the radial distribution functions, *g*(*r*) (Figure S6a). Another widely used correlation function is the reduced pair distribution function, *G*(*r*) = 4*πrρ_0_*[*g*(*r)* ­– 1]. As *r*→0 *G*(*r*) behaves like -*4πrρ_0_,* which is a straight line going through zero with a slope that is proportional to the average number density of the material. In Figure S6b, the slope was calculated for amorphous AuGa_2_ at ambient conditions, and yields an atomic number density 0.0432 (± 0.016) atoms/Å^3^ at room temperature and zero pressure.

The second method used experimental values from the refined unit cell volumes of the crystalline phases slightly before melting (Figure S6c). The values in Table 2 in the main text are determined using the latter method. Table S1 compares the atomic number densities calculated using the two different methods.

**Table S1**

Atomic number densities determined using two different methods.

| P(GPa) | T(K) | From the slope of atomic distribution (atoms/Å^3^) | from the refined unit cell volumes of the crystalline phases slightly before melting (atoms/Å^3^) |
| --- | --- | --- | --- |
| 0 | 300 | 0.043 ± 0.016 | 0.0535 |
| 1.5 | 803 | 0.046 ± 0.022 | 0.0547 |
| 6.3 | 823 | 0.050 ± 0.018 | 0.0577 |
| 5.5 | 863 | 0.046 ± 0.039 | 0.0571 |
| 11 | 863 | 0.053 ± 0.018 | 0.0595 |
| 16.2 | 938 | 0.062 ± 0.008 | 0.0615 |


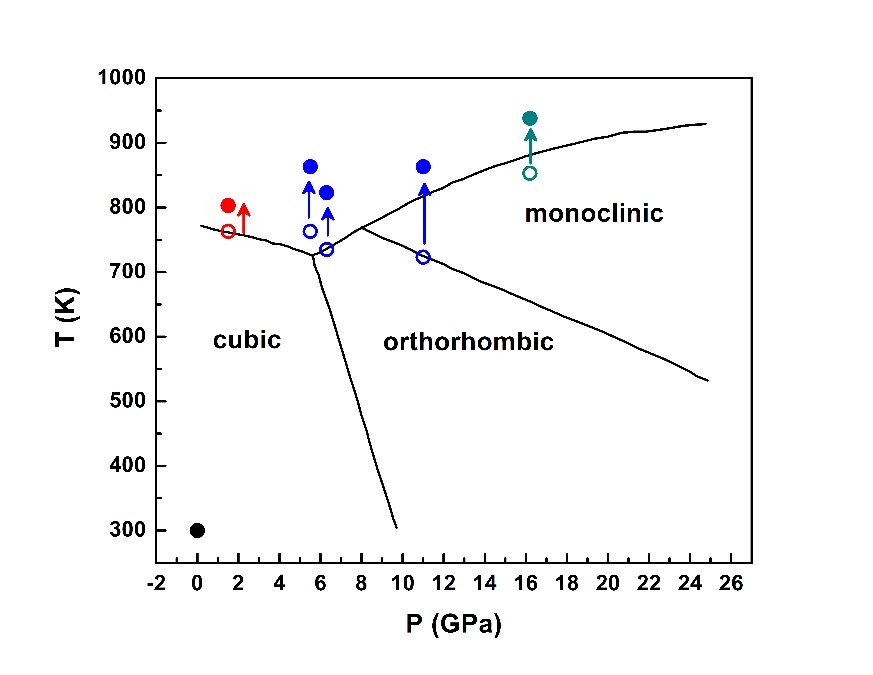


**(c)**


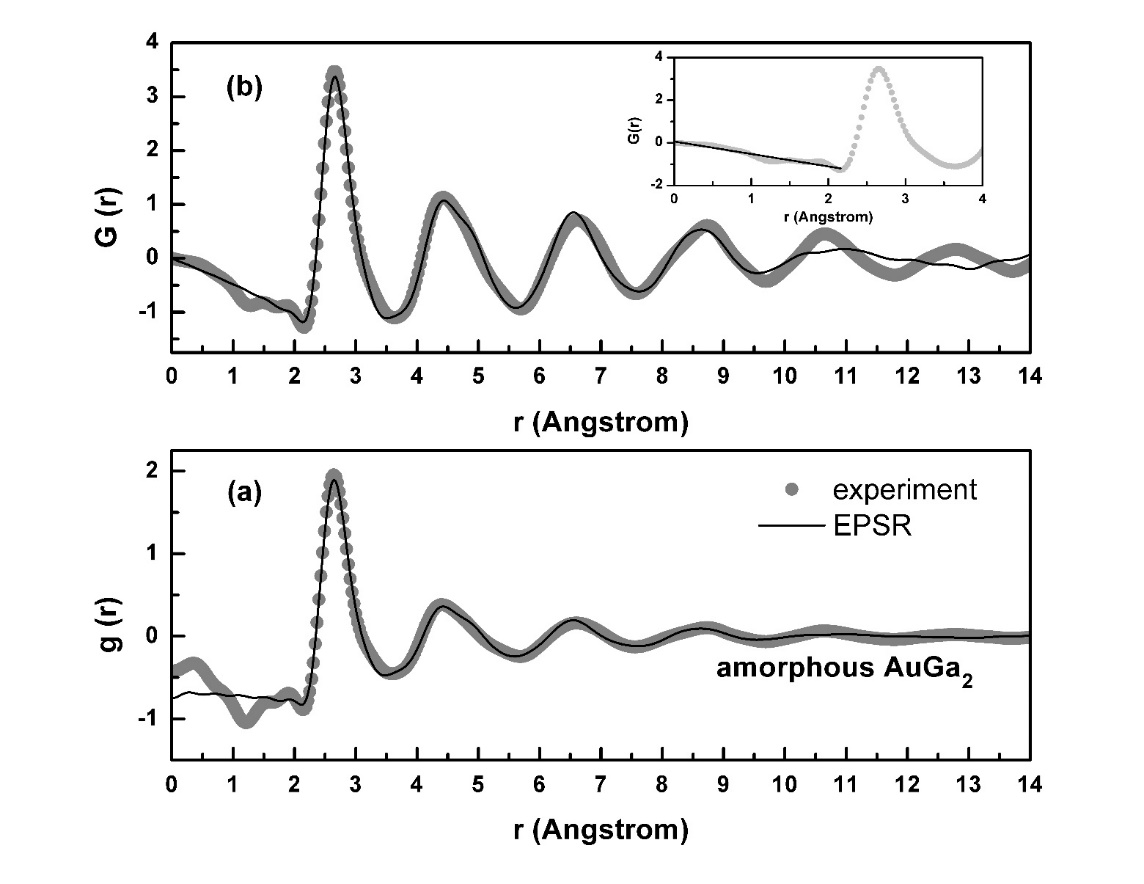


**Slope = - 4π*ρ_0_***

**Figure S6.** (a) Experimental and calculated atomic distribution function, *g*(*r*), showing the major interatomic distances in amorphous AuGa_2_. (b) Reduced pair distribution function, *G*(*r*), and the linear fit below 2.2 Å giving the slope that is used to calculate the atomic number density of amorphous AuGa_2_ at ambient conditions. (c) Phase diagram of AuGa_2_ (from ref. [14]). Filled circles represent the temperature–pressure values at which total structures factors were obtained in the present study. Open circles represent the thermodynamic conditions at which the atomic number densities of the related crystalline phases were calculated.

1. Data reduction to get *S*(*Q*) from raw XRD patterns

The experimentally measured x-ray intensities, *I^obs^(Q)* can be expressed as the sum of scattering from the sample, *I^samp^(Q)* and background, *I^back^(Q)* :

*I^obs^ (Q)=T(Q) I^samp^(Q) + b I^back^(Q)*$I^{\mathrm{obs}}\left( Q \right)=T\left( Q \right) I^{\mathrm{samp}}\left( Q \right)+bI^{\mathrm{back}}\left( Q \right)$ (3)

where *T(Q)* is the absorption correction*,* which is a function of the scattering angle, and *b* is the background correction factor. *T(Q)* was computed based on our DAC geometry, the absorption coefficients of diamond anvils, and conventional backing plates using software package *Absorb6.0* [2]. The sample self-absorption is usually small for thin DAC samples (< 50µm thickness), so is neglected. Bragg diffraction peaks from diamond and gasket were masked within FIT2D [3] before performing the angular integrations*.* Proper background subtraction is a challenge in structural studies of non-crystalline materials with DAC experiments, and needs to be carefully determined. Scattering from air, pressure medium and diamond anvils contribute to the background, and can be affected by variations in incident-beam intensity, detector temperature, and other time-dependent factors. Background correction factors, *b*, were computed at each pressure using the iteration method of Eggert *et. al*. [4]. The description of the atomic distribution in non-crystalline materials usually employs the concepts of the structure factor and pair distribution functions in atomic units. By introducing a normalization factor *N*, the total scattering from the sample *I^samp^*(*Q*) can be expressed in atomic units in terms of coherent scattering *I^coh^(Q)*, incoherent scattering *I^incoh^(Q)*, and multiple scattering *I^mul^(Q)*:

*N I^samp^(Q)=I^coh^(Q) + I^incoh^(Q) + I^mul^(Q)*$\mathrm{NI}^{\mathrm{samp}}\left( Q \right)=I^{\mathrm{coh}}\left( Q \right)+I^{\mathrm{incoh}}\left( Q \right)+I^{\mathrm{mul}}\left( Q \right)$ (4)

The incoherent scattering (Compton scattering) contribution can be computed using the analytic approximation reported by Cromer and Mann [5], and Cromer [6]:

*I^incoh^(s) = M [1 - (1+as^2^+bs^4^)(1+cs^2^+ds^4^)^-2^]* (5)

where *M* = *Z* (atomic number) for neutral atoms, *s* = *λ^-1^sinθ* = *Q/4π* and *a, b, c* and *d* are non-linear least square fit parameters. Multiple scattering is relatively weak, and neglected in our high-pressure x-ray scattering studies. The measured intensities were converted into electron units per atom by the generalized Krogh-Moe-Norman method [7], using the atomic scattering factors, *f(Q)* tabulated in the International Tables [5, 6]. The coherent x-ray scattering intensity, *I^coh^(Q)* for a binary disordered system is expressed by the following in terms of the Faber-Ziman (FZ) notation,

*I^coh^(Q) = (< f ^2^>* – *< f > ^2^) + < f > ^2^ S(Q)* (6)

The *< f ^2^ >* ­– *< f > ^2^* t$\left\langle f^{2} \right\rangle$erm is attributed to the intensity arising only from the difference in the atomic scattering factors of the constituent atoms. The square of the mean scattering factor *< f >^2^* and the mean square average of the scattering factor *<f ^2^>* were determined using the atomic fractions of Au and Ga in the AuGa_2_ binary system [8]. A typical example of the collected and processed diffraction intensities is shown in Figure S7. The reproducibility of these calculations was confirmed with multiple runs and samples. The total structure factors, *S*(*Q*) were obtained from the experimental *I(Q)* data by way of Equations 3-6, using the software package PDFGETX2 [9].

**
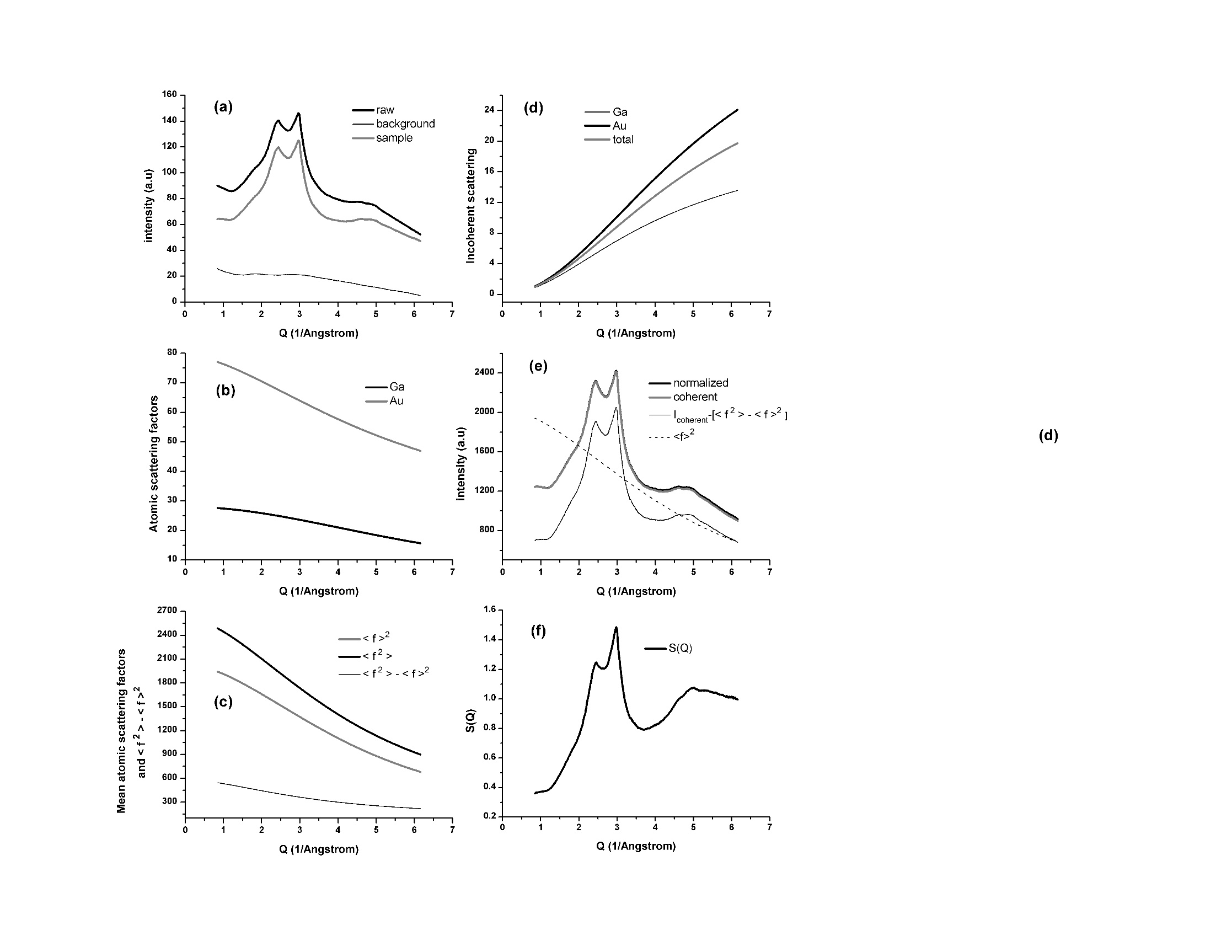
**

**Figure S7.** Details of the data reduction process to obtain the structure factor *S*(*Q*) from the melt phase at 11 GPa and 863 K. (a) Raw (*thick black solid line*) and background (*thin black solid line*) scattering collected from the sample and sample cavity. Also shown is the pattern after background and absorption corrections (*thick grey solid line*). Atomic scattering factors (b, c), incoherent scattering (d) and normalized data (e) are also shown. (f) Total structure factor of liquid AuGa_2_.

1. Capability of EPSR technique and quality of *S*(*Q*) data for high-pressure melt; cross check with high-quality *S*(*Q*) data from amorphous AuGa_2_ at zero pressure

We compare the EPSR simulations with experimental data of Bohorquez et al. [10] for amorphous AuGa_2_ at zero pressure in Figure S8. The *Q_max_* is 14 Å^−1^ (Figure S8a) corresponding to a resolution in *g*(*r*) of *Δr* ~ 0.448 Å. This high-quality *S*(*Q*) pattern for the amorphous phase not only enables us to understand the capability of EPSR simulations, but also gives us a chance to optimize the set of refinement constraints as given in the main text. As with previous studies [11, 12], it is recognized that EPSR and Reverse Monte Carlo (RMC) are not intended to compete with theoretical computer simulations, such as classical molecular dynamics (MD) and density functional theory molecular dynamic (DFT-MD) simulations. Rather, EPSR and RMC are complementary to the theoretical methods in offering a means of fitting experimental data. The melting temperature of AuGa_2_ decreases up to $\sim$6 GPa [13, 14], reversing slope above this pressure due to structural phase transition [14], which makes it difficult to obtain reliable potential parameters for classical-MD. DFT-MD requires large simulations for generating reliable melt structure factors, demanding considerable computing. The crystal-structural transitions, cubic → orthorhombic → monoclinic observed experimentally in AuGa_2_ with increasing pressure are explained satisfactorily by DFT calculations [15].


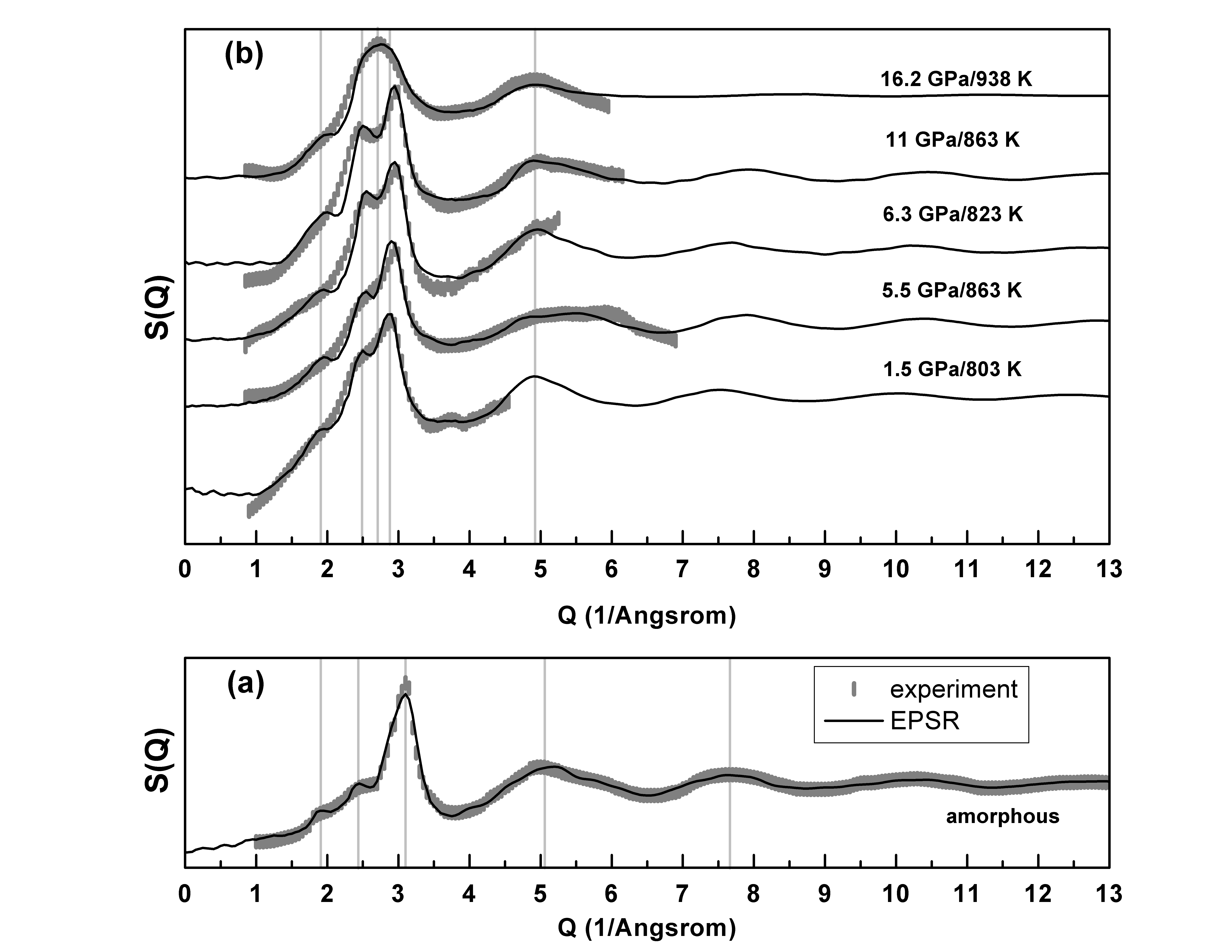


**Figure S8.** X-ray scattering data. Experimental (*thick grey bars*) and EPSR simulated (*black thin lines*) total structure factors, *S*(*Q*) for (a) amorphous AuGa_2_ at zero pressure [10], and (b) for liquid AuGa_2_ at high pressures. Thin grey lines are for comparison of the peak positions in the *S*(*Q*) patterns.

While data collected with higher energy x-rays give improved results, it is possible to obtain reasonable partial structure factors and corresponding partial pair distribution functions with the measurements presented here, which include the first liquid structure data at high pressures for AuGa_2_ starting in the orthorhombic cotunnite phase. This is an analog for liquid metallic SiO_2_ at conditions for which the cotunnite structure is stable, above 600 GPa [16].

1. Number of iterations and the quality of *S*(*Q*) fits


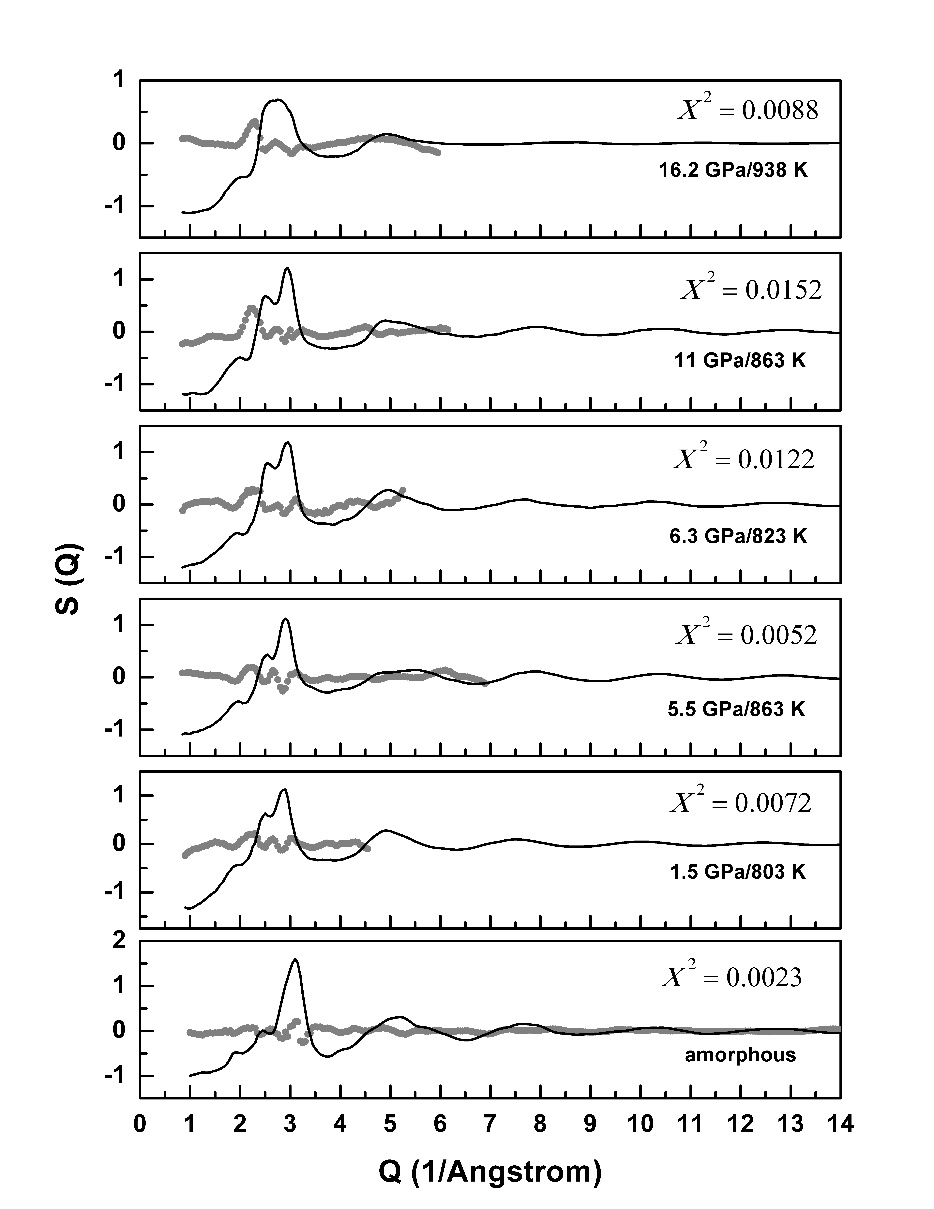


**Figure S9.** EPSR fits to x-ray diffraction data from liquid AuGa_2_ (*black lines*), and residual between the data and fit (*grey dots*). Corresponding *χ*^2^ values for the EPSR simulations are given for each pattern.

Figure S9 shows fits to the liquid-AuGa_2_ diffraction data for the proposed model, and the residual between fit and data. The largest contribution to the *χ*^2^ values comes from the difference between fit and data in the high-*Q* region of the present study.

1.
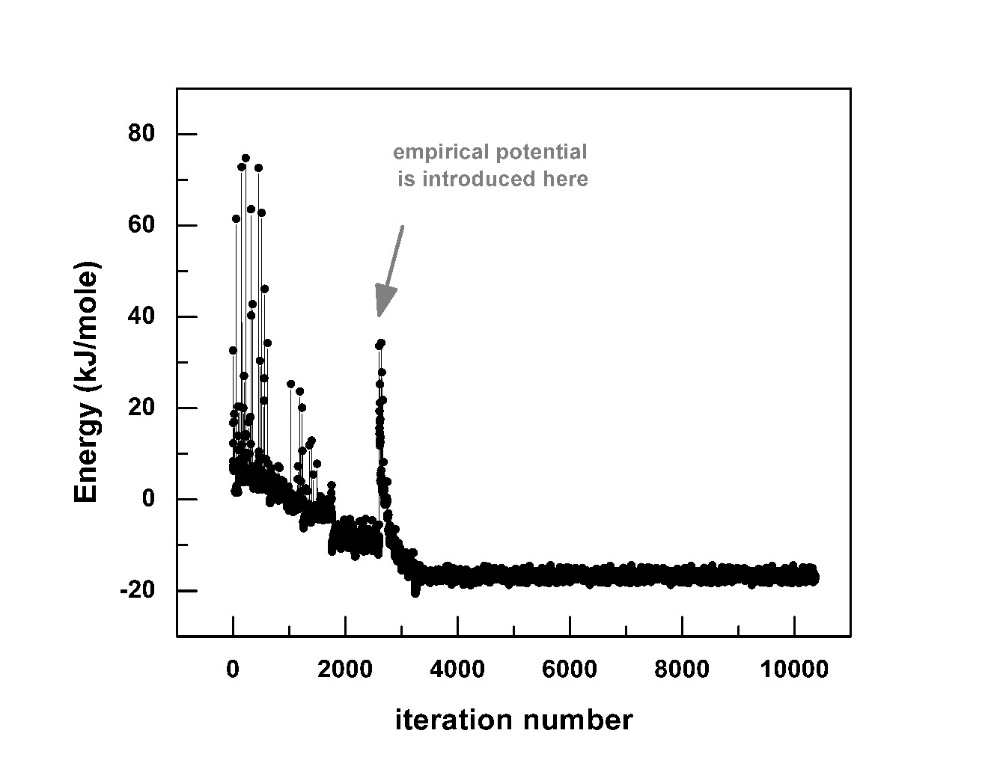
Other supporting figures

**Figure S10.** Energy at each iteration of the simulation.


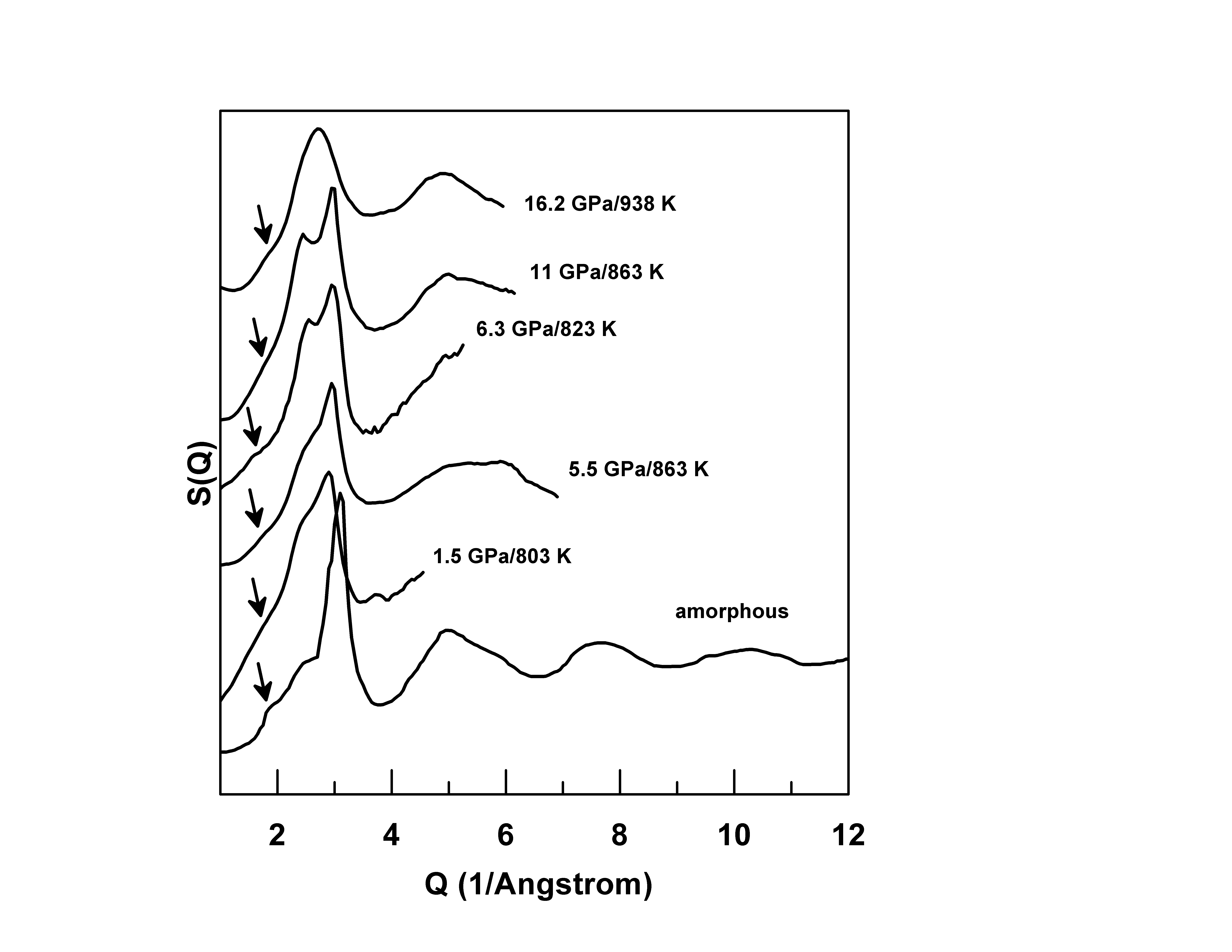


**Figure S11.** Experimental total structure factors *S*(*Q*) for amorphous-AuGa_2_ and melt-AuGa_2_. Arrows indicate the position of the first sharp diffraction peak (FSDP).


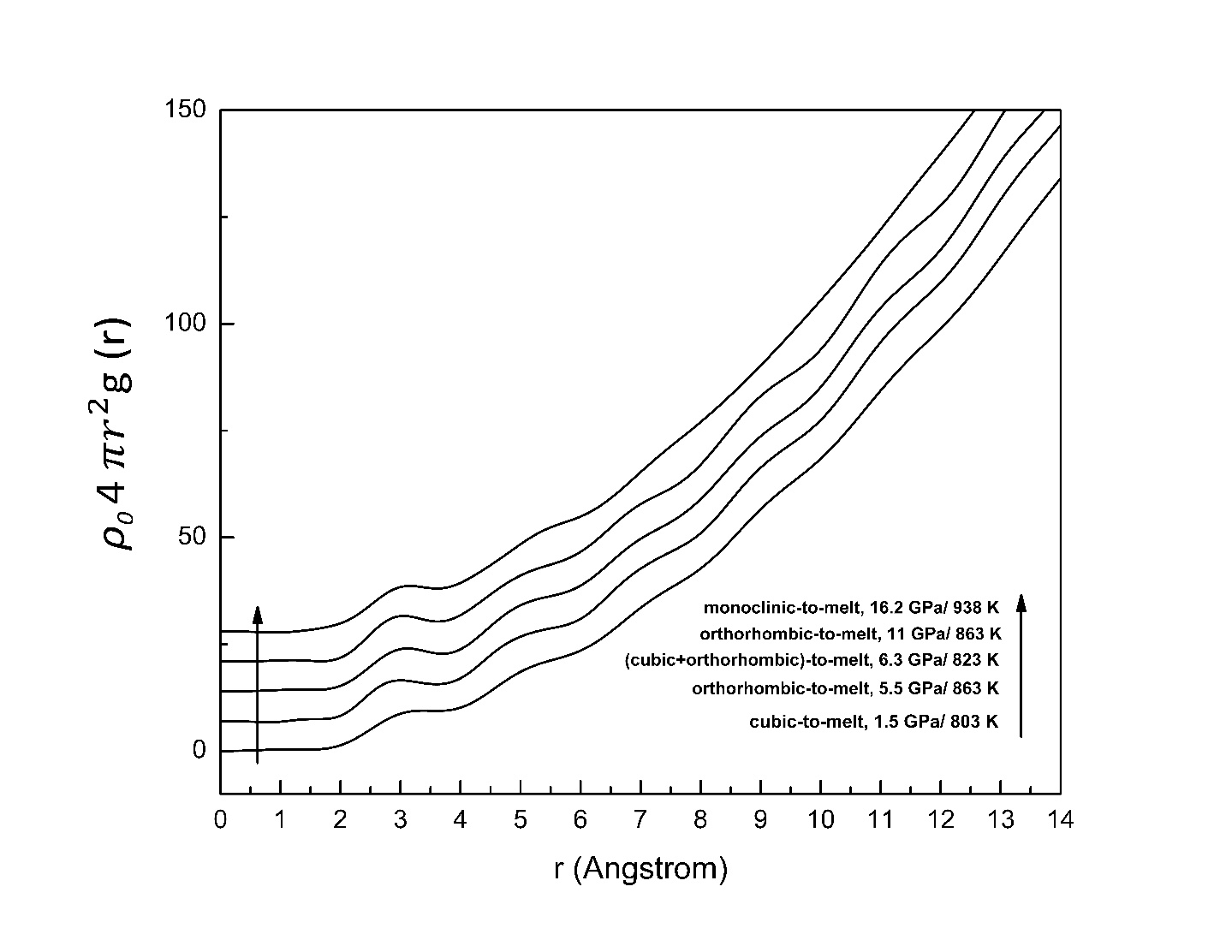


**Figure S12.** Radial distribution functions, *g*(*r*) in spherical coordinates.

**References used in supplementary information:**

### [1] Fawcett R W 1973 *Calc. Tiss. Res.* **13** 319-325.

### [2] Angel R J 2004 *J. Appl. Cryst.* **37** 486-492.

# [3] Hammersley A P, Svensson S O, Hanfland M, Fitch A N, Hausermann D 1996 *High Pressure Research* **14** 235.

[4] Eggert J H, Weck G, Loubeyre P, Mezouar M 2002 *Phys. Rev. B* **65** 174105.

### [5] Cromer D T, Mann J B 1967 *J. Chem. Phys.* **47** 1892-1893.

[6] Cromer D T 1969 *J. Chem. Phys.* **50** 4857-4859.

[7] Krogh-Moe J A 1956 *Acta Cryst.* **9** 951.

[8] Balyuzi H H M 1975 *Acta Cryst. A* **31** 600.

### [9] Qiu X, Thompson J W, Billinge S J L 2004 *J. Appl. Crystallogr.* **37** 678.

[10] Bohórquez A 1991 *Revista Mexicana de Física*. **37** (2) 268-275.

[11] McGreevy R L 2001 *J. Phys.: Condens. Matter* **13** R877–R913.

[12] Soper A. K. 2007 *J. Phys.: Condens. Matter* **19** 415108.

[13] Storm AR, Wenick J H and Jayaraman A 1966 J. Phys. Chem. Solids. **27** 1227.

[14] Geballe Z M, Raju S V, Godwal B K, Jeanloz R 2013 *J. Phys.:Cond. Matter* **25** 415401.

[15] Garg *et al.* 2006 *J. Phys.: Condens. Matter* **19** 425224.

[16] Godwal *et al.* 2013 *Phys. Rev. B* **87** 100101R.
